# Toward a monophyletic *Cheilanthes*: The resurrection and recircumscription of *Myriopteris* (Pteridaceae)

**DOI:** 10.3897/phytokeys.32.6733

**Published:** 2013-12-19

**Authors:** Amanda Lee Grusz, Michael Dennis Windham

**Affiliations:** 1 Department of Biology, Duke University, Box 90338, Durham, NC 27708–0338, USA

**Keywords:** *Cheilanthes*, cheilanthoid, myriopterid, myriopteroid, nomenclature, taxonomy

## Abstract

The fern genus *Cheilanthes* (Pteridaceae) has perplexed taxonomists for more than two centuries. Complex patterns of evolution involving rampant morphological convergence, polyploidy, hybridization, and apomixis have made the taxonomy of this group especially difficult. Fortunately, recent phylogenetic analyses have helped to clarify relationships among cheilanthoid taxa. Based on these findings, we here formalize an updated taxonomy for one monophyletic clade comprising 47 primarily North and Central American taxa usually included in *Cheilanthes*. Because the type species of *Cheilanthes* (*Cheilanthes micropteris*) is only distantly related to this clade, we resurrect the genus *Myriopteris* to accommodate these taxa, and present a revised circumscription for the group, including 36 new combinations.

## Introduction

A “practical and natural” generic classification of cheilanthoid ferns (Pteridaceae) has eluded taxonomists for more than 200 years and was viewed by Tryon and Tryon (1982) as one of the most contentious issues in fern systematics. Central to the problem is the circumscription of the large genus *Cheilanthes*, which all molecular studies with sufficient sampling indicate is polyphyletic (see Gastony and Rollo 1998; Kirkpatrick 2007; Prado et al. 2007; Schuettpelz et al. 2007; Zhang et al. 2007; Rothfels et al. 2008; Eiserhardt et al. 2011). Since the initial description of *Cheilanthes* (Swartz 1806) encompassing 16 species, various authors have moved hundreds of taxa into (e.g., Domin 1913; Mickel 1979) and out of (e.g., Fée 1852; Smith 1875; Ching 1941) the genus. Of the ca. 500 validly published species names in *Cheilanthes*, some 60% have, at some point, resided in other genera. The lack of definitive taxonomic characters in this group often is attributed to widespread convergent evolution in the drought-prone habitats occupied by these ferns (Tryon and Tryon 1973, 1982), and the problem is likely insoluble based on morphology alone. However, the same genetic evidence that highlights shortfalls in the current classification provides a key to solving this puzzle. As DNA sequence data proliferate and morphological features are reexamined in light of molecular phylogenies, it eventually becomes possible to recognize monophyletic assemblages of species that can be circumscribed as genera. We now have reached this point with certain groups of cheilanthoid ferns, at least in terms of removing taxa and clades that cannot reasonably be included within *Cheilanthes* (Link-Perez et al. 2011; Li et al. 2012).

**Figure 1.**
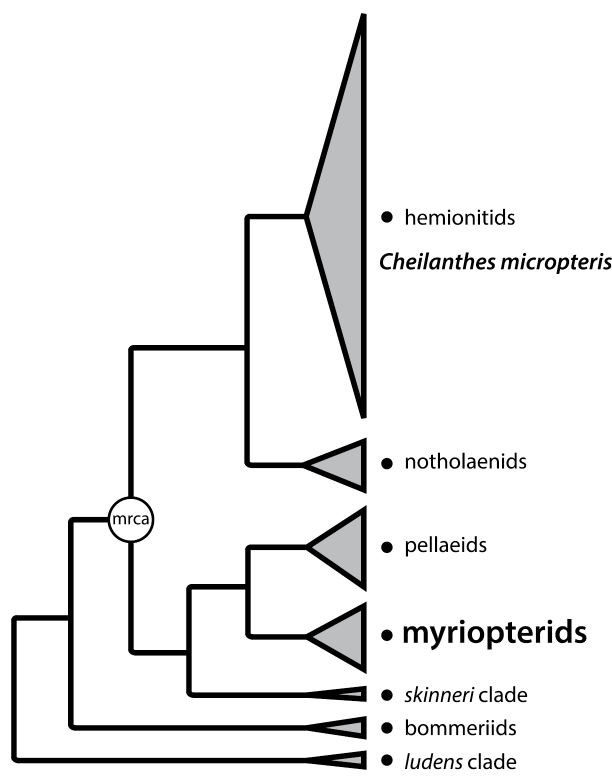
Summary phylogeny for cheilanthoid ferns, indicating the placement of *Cheilanthes micropteris* (the type species for *Cheilanthes*) within the hemionitid clade—only distantly related to the myriopterid clade. The six major clades of cheilanthoid ferns are shown with tips roughly proportional to clade size. The most recent common ancestor (mrca) of *Cheilanthes micropteris* and the myriopterid clade is indicated. Modified with permission from Windham et al. (2009).

Here, we focus on the primarily New World lineage previously referred to as the “American *Cheilanthes*” (Kirkpatrick 2007), myriopteroid (Rothfels et al. 2008), or myriopterid (Windham et al. 2009; Eiserhardt et al. 2011) ferns. Limited sampling in each of those analyses indicated that these ferns might represent a well-supported, monophyletic group, an assumption fully supported by the more complete (85%) taxon sampling of Grusz et al. (in review). In addition to suggesting the monophyly of the myriopterid lineage, the analyses of Rothfels et al. (2008) and Eiserhardt et al. (2011) conclusively demonstrated that this clade was quite distantly related to the type species of *Cheilanthes*, *Cheilanthes micropteris* (results summarized in Fig. 1). This improved understanding of phylogenetic relationships among cheilanthoid ferns necessitates a taxonomic revision that can be achieved by one of two options: 1) all taxa derived from the most recent common ancestor of *Cheilanthes micropteris* and the myriopterid ferns could be assigned to a single genus (which would not be called *Cheilanthes* because of the priority of *Hemionitis*), or 2) myriopterid ferns could be transferred to a different genus, reflecting their phylogenetic distinction from *Cheilanthes* s.s. The first option would require 400+ new combinations in *Hemionitis* (or the conservation of *Cheilanthes* against it followed by more than 100 new combinations in that genus). It would also subsume a number of cohesive, well-characterized genera that are clearly distinct based on morphological, molecular, and cytological grounds, including *Adiantopsis* (Link-Pérez et al. 2011), *Argyrochosma* (Windham 1987; Sigel et al. 2011), *Astrolepis* (Beck et al. 2010), *Doryopteris* (Yesilyurt 2004), *Gaga* (Li et al. 2012), and *Notholaena* (Rothfels et al. 2008). This approach would maximize the number of nomenclatural changes while simultaneously obscuring well-documented phylogenetic relationships, resulting in the inclusion of all but six cheilanthoid species in one genus. Because we consider this option untenable, we have, instead, chosen to remove the myriopterid ferns from *Cheilanthes*.

When any species or clade is removed from *Cheilanthes*, the first issue that must be addressed involves their relationship to *Allosorus pusillus* (Willd. ex Bernh.) Bernh. [= *Cheilanthes pteridioides* (Reich.) C. Chr.]. This species was designated the lectotype of *Allosorus* Bernh. by Pichi-Sermolli (1953), a choice subsequently validated by the ICBN when *Cheilanthes* was conserved over *Allosorus* (Appendix II of the Montreal Code, Stafleu et al. 1961). The only phylogenetic study published to date that includes the type species of both *Allosorus* and *Cheilanthes* is that of Eiserhardt et al. (2011). In that analysis, it is unclear whether the divergence between *Cheilanthes maderensis* (= *Cheilanthes pteridioides*; see Nardi and Reichstein 1985; Rothfels et al. 2012) and *Cheilanthes micropteris* is sufficient to justify the recognition of two genera. The two taxa appear in distinct, well-supported clades (clade A vs. clade C in fig. 2B of Eiserhardt et al. 2011), but deeper relationships are poorly resolved and both clearly belong to the rapidly diversifying hemionitid lineage (clade H). The unequivocal assignment of *Allosorus* to the hemionitids by Eiserhardt et al. (2011) does, however, prevent the application of this generic name to the myriopterid clade. Any attempt to expand *Allosorus* to include myriopterids would encompass both *Cheilanthes* (conserved over *Allosorus*) and *Hemionitis* (which has priority over both).

One potentially viable option for generic placement of the myriopterid clade would be to include it within a revised circumscription of *Pellaea* Link. All recent phylogenetic studies with adequate sampling of the two groups (e.g., Kirkpatrick 2007; Rothfels et al. 2008; Eiserhardt et al. 2011) strongly support the position of myriopterids as the sister group of the pellaeid clade, which includes *Pellaea atropurpurea*, the lectotype of the oldest generic name applicable to that clade. We are not in favor of expanding the definition of *Pellaea* to encompass the myriopterids for a variety of reasons. First, the two are quite distinct, both in terms of phylogenetic divergence and morphology. The myriopterids have substantially smaller ultimate segments, pubescent and/or scaly (vs. mostly glabrous) leaf blades, and sporangia that are confined to vein tips (vs. distributed along the veins near the segment margins). Because of these differences, the two groups generally have not been considered closely related, and most myriopterids would require new combinations in *Pellaea*. Adding to this nomenclatural upheaval is the fact that other well-defined genera, including *Argyrochosma* (Sigel et al. 2011) and *Astrolepis* (Beck et al. 2010), would be subsumed within such a circumscription of *Pellaea*, which would require additional new combinations and serve only to further undermine the distinctions among the major genera of cheilanthoid ferns.

If the expansion of *Pellaea* is ruled out, there remain three other generic names typified by species belonging to the myriopterid clade: 1) *Myriopteris*, described by Fée (1852) and typified by *Myriopteris marsupianthes* Fée; 2) *Cheilosoria*, named by Trevisan (1877) and lectotypified by Copeland (1947) based on *Cheilosoria allosuroides* (Mett.) Trev.; and 3) *Pomataphytum*, published by Jones (1930) and typified by *Pomataphytum pocillatum* M. E. Jones(= *Myriopteris lendigera*). Phylogenetic reconstructions (Grusz et al. in review) confirm that the type species of *Myriopteris* and *Pomataphytum* fall within a single, well-supported clade. In fact, the diploid species *Myriopteris marsupianthes* is thought to be one of the parents of sexual tetraploid *Myriopteris lendigera* (see Mickel and Smith 2004). Thus, the generic name *Pomataphytum* is appropriately considered a taxonomic synonym of the earlier described *Myriopteris* and can be eliminated as a potential name for the myriopterid clade. Copeland’s (1947) lectotype of *Cheilosoria* belongs to the well-supported and morphologically distinctive alabamensis clade that diverges earlier in the myriopterid phylogeny (Grusz et al. in review), and the name *Cheilosoria* could be used for this particular group if the myriopterids were subdivided into two or more genera. However, *Myriopteris* predates *Cheilosoria* by 25 years and, when these species are assigned to a single genus (our preferred approach), *Myriopteris* is the correct generic name for the inclusive myriopterid clade.

## Historical use of the name *Myriopteris*

The original concept of *Myriopteris* (Fée 1852) included 11 species, these split between two sections (*Eumyriopteris* and *Cheilanthastrum*) distinguished by the presence or absence of a well-developed, inframarginal false indusium. The Latin and French descriptions of the genus are only partly overlapping; shared elements include the highly divided fronds, the small, orbicular ultimate segments with recurved margins (“formant un bourrelet très-contracté”), and a tendency to be covered by hairs and/or scales. *Myriopteris* was accepted and significantly expanded by J. Smith (1875: 280) who stated “the genus consists of about 20 species, distinguished from *Notholaena* and *Cheilanthes* by their small, concave, lenticular segments.” The segregation of *Myriopteris* from *Cheilanthes* was, however, rejected by most subsequent authors (e.g., Christensen 1906; Copeland 1947; Lellinger 1965; Tryon and Tryon 1982; Kramer et al. 1990), with two notable exceptions. Pichi-Sermolli (1977) advocated a narrowed circumscription of the genus, including only the two species with prominent false indusia, viz., *Myriopteris marsupianthes* and *Myriopteris lendigera*. As shown by Grusz et al. (in review), this definition of *Myriopteris* is phylogenetically indefensible because it excludes *Myriopteris mexicana*, the apparent maternal progenitor of allotetraploid *Myriopteris lendigera*. About the same time Pichi-Sermolli was narrowing the definition of *Myriopteris*, Löve and Löve (1977) expanded it slightly by proposing a new combination for the species known as *Cheilanthes covillei* Maxon. This was done without explanation, though almost certainly reflects the fact that this species has the small, bead-like ultimate segments emphasized in earlier circumscriptions of the genus.

Although this “microphyllous” leaf morphology is common within *Myriopteris*, it does not characterize the entire clade (Grusz et al. in review) and has evolved independently in other cheilanthoid lineages. Thus, the possession of small, bead-like ultimate segments does not constitute a synapomorphy for the genus as defined herein. In fact, our list of excluded names (see Taxonomic Treatment) includes seven taxa with bead-like segments previously ascribed to *Myriopteris* but more closely related to *Cheilanthes* s.s. (Windham et al. unpublished). Because all morphological characters used by previous authors to define *Myriopteris* are subject to strong, positive selection in xeric-adapted cheilanthoid lineages (Hevly 1963), it is not surprising that none of them uniquely define the genus. The totality of evidence, however, indicates that the myriopterids represent a deeply divergent clade that cannot reasonably be combined with any other in a single genus. Therefore, we propose to resurrect *Myriopteris* and recircumscribe it to encompass the entirety of this well supported cheilanthoid lineage.

### Distinguishing *Myriopteris* Fée emend. Grusz & Windham from *Cheilanthes* s.s.

Ideally, morphological and/or cytological synapomorphies would substantiate phylogenetic relationships inferred from DNA sequence data. However, easily observed synapomorphies distinguishing the various clades of cheilanthoid ferns are few, and homoplastic characters abound. To paraphrase Sir William Hooker (1852: 75), “Vain is the attempt to form a definite character which shall decide the limits of [*Cheilanthes*],” a statement that applies equally well to *Myriopteris*. Highly divided (decompound) leaf blades with small ultimate segments are scattered across the cheilanthoid tree and, indeed, are characteristic of ferns in general, and an indument of hairs and/or scales is one common strategy among plants used to reduce water loss in xeric habitats (Hevly 1963). Other characters useful for species-level identification within myriopterids, such as vernation, are, without exception, shared with other distantly related cheilanthoid ferns.

Molecular analyses spanning the diversity of cheilanthoid species (Windham et al. unpublished) illuminate one particularly useful character distinguishing *Myriopteris*, as defined herein, from *Cheilanthes* s.s. The taxa most closely related to the type species of the latter [*Cheilanthes micropteris* plus all Australian *Cheilanthes* and a group of South American species including the *Cheilanthes scariosa* (Sw.) C. Presl complex of Tryon and Tryon (1982), *Cheilanthes obducta* Mett. ex Kuhn, and *Cheilanthes fractifera* R. M. Tryon] have 32 small spores per sporangium when sexual, and 16 large spores per sporangium when apomictic. This intriguing cytological synapomorphy results from the elimination of a premeiotic mitosis in the cell lineages generating the sporocytes (Windham et al. unpublished). Aside from a few species of the distantly related genus *Notholaena*, all other cheilanthoid ferns so far examined (including every *Myriopteris* species; Grusz et al. in review) produce 64 small spores per sporangium in sexual individuals and 32 large spores per sporangium in apomicts. This character appears to provide an absolute separation between *Myriopteris* and *Cheilanthes* s.s., and is easily observed using a dissecting microscope. In combination with differences in spore ornamentation (see Tryon and Lugardon 1991), leaf venation (Pryer et al. 2010), and geographic distribution, this feature provides a clear distinction between the two genera. For diagnostic purposes, then, *Myriopteris* Fée emend. Grusz & Windham differs from *Cheilanthes* s.s. (i.e., *Cheilanthes micropteris* and its close relatives) in its production of 64 small or 32 large (vs. 32 small or 16 large) spores per sporangium; mostly cristate or rugulose (vs. echinate, granulose, or verrucate) spore ornamentation; a lack of obvious vein endings near the margins of the ultimate segments (vs. often prominent hydathodes), and a largely North and Central American (vs. exclusively South American/Old World) distribution.

## Taxonomic treatment

### Myriopteris

Fée emend. Grusz & Windham

http://species-id.net/wiki/Myriopteris

#### Type.

*Myriopteris marsupianthes* Fée, Mém. Fam. Foug. 5: 149, t. 12A. f. 1. 1852

#### Description.

Plants rupestral or terrestrial. Rhizomes compact to long-creeping, ascending or horizontal, scaly. Rhizome scales lanceolate to acicular, concolorous (tan to dark brown) or bicolorous (with dark central stripe and brown margins). Leaf vernation non-circinate to circinate. Petiolescastaneous to black, scaly and/or pubescent, rarely almost glabrous. Rachises terete or flattened or grooved adaxially, with indument similar to that of the petioles. Blades2- to 4-pinnate (rarely pinnate-pinnatifid), lanceolate to ovate-deltate, occasionally linear or pentagonal; adaxial surfaces glabrous or pubescent; abaxial surfaces scaly and/or pubescent or rarely glabrous. Ultimate segments round to oblong-ovate, minute to >1 cm long, the veins obscure and not ending in prominent hydathodes. Segment margins usually recurved, with a poorly differentiated false indusium (strongly differentiated in *Myriopteris lendigera* and *Myriopteris marsupianthes*). Sori usually partly to completely covered by the recurved segment margins, the sporangia clustered at vein tips. Sporangia 64-spored (in sexual species) or 32-spored (in apomicts). Spores globose-tetrahedral, tan to brown, cristate to rugulate. Chromosome numbers *n* = 29, 30, 58, 60 (sexual species); *n* = 2*n* = 87, 90 (apomictic triploids); *n* = 2*n* = 120 (apomictic tetraploids).

#### Distribution.

Species of *Myriopteris* range from southern Canada through the Caribbean and Central America to southern Chile, with one species (*Myriopteris rawsonii*) endemic to Namibia and South Africa. Mexico is the center of species diversity for the genus; 34 of the 44 species can be found in Mexico, and seven of these are endemic.

##### New and resurrected combinations in *Myriopteris*

**1)** *Myriopteris aemula* (Maxon) Grusz & Windham, **comb. nov.** *Cheilanthes aemula* Maxon, Contr. U.S. Natl. Herb. 10: 495. 1908. Type: Mexico. Tamaulipas: Victoria, in river canyon, under overhanging rocks, altitude about 320 meters, February 1 to April 9, 1907, Palmer 187 (holotype: US; isotype: US). urn:lsid:ipni.org:names:77134841-1

**2)** *Myriopteris alabamensis*(Buckley) Grusz & Windham, **comb. nov.** *Pteris alabamensis* Buckley, Amer. J. Sci. Arts 45: 177. 1843. *Cheilanthes alabamensis* (Buckley) Kunze, Linnaea 20: 4. 1847. Type: USA. Alabama: Growing in tufts on limestone rocks that form the banks of the Tennessee River, at the foot of Muscle Shoals, Buckley s.n. (holotype: PH; isotypes: MO, NY). urn:lsid:ipni.org:names:77134842-1

**3) ***Myriopteris allosuroides* (Mett.) Grusz & Windham, **comb. nov.** *Cheilanthes allosuroides* Mett., Abh. Senckenberg. Naturf. Ges. 3: 78. 1859. *Pellaea allosuroides* (Mett.) Hieron., Hedwigia 62: 18. 1920. Type: Mexico, Schmitz s.n. (holotype: location unknown). urn:lsid:ipni.org:names:77134843-1

**4)** *Myriopteris aurea* (Poir.) Grusz & Windham, **comb. nov.** *Pteris aurea* Poir. Encyclopédie Méthodique, Botanique 5: 710. 1804. Type: Peru. Elle a été recueillie au Pérou par Joseph de Jussieu s.n. (sheet 1333 in hb. Jussieu; holotype: P). urn:lsid:ipni.org:names:77134844-1

* Acrostichum bonariense* Willd., Sp. Pl., ed. 4, 5(1): 114. 1810. *Notholaena bonariensis* (Willd.) C. Chr., Index Filic. 459. 1906. *Cheilanthes bonariensis* (Willd.) Proctor, Bull. Inst. Jamaica, Sci. Ser. 5: 15. 1953.

 In *Cheilanthes*, this has been called *Cheilanthes bonariensis* (Willd.) Proctor because use of the oldest applicable epithet (based on *Pteris aurea* Poir.) was blocked by the earlier publication of *Cheilanthes aurea* Baker (Proctor 1953). With the transfer of this species to *Myriopteris* we revert to the older epithet and thus avoid the typification difficulties associated with the basionym *Acrostichum bonariense* Willd. (Ponce and Zimmer 2011).

**5)** *Myriopteris chipinquensis* (Knobloch & Lellinger) Grusz & Windham, **comb. nov.** *Cheilanthes chipinquensis* Knobloch & Lellinger, Amer. Fern J. 59: 8. 1969. Type: Mexico. Nuevo Leon: Chipinque Mesa, outside Monterey, Knobloch 1996B (holotype: MSC; isotypes: F, GH, MEXU, MICH, UC, US). urn:lsid:ipni.org:names:77134845-1

**6)** *Myriopteris cinnamomea*(Baker) Grusz & Windham, **comb. nov.** *Notholaena cinnamomea* Baker in Hook. & Baker, Syn. Fil. ed. 2. 515. 1874. *Cheilanthes cinnamomea* (Baker) Domin., Biblioth. Bot. 20: 133. 1913. *hom. illeg. non Cheilanthes cinnamomea* D. C. Eaton, Proc. Amer. Acad. Arts 18: 186. 1883. Type: Guatemala. Mo[n]tagua, 1862, Salvin & Goodman s.n. (holotype: K; isotype: BM). urn:lsid:ipni.org:names:77134870-1

* Cheilanthes tryonii* T. Reeves, Brittonia 32: 504. 1980.

 In *Cheilanthes*, this species has been called *Cheilanthes tryonii* T. Reeves because use of the oldest applicable epithet (based on *Notholaena cinnamomea* Baker) was blocked by the earlier publication of *Cheilanthes cinnamomea* D. C. Eaton (Reeves 1980). With the transfer of this species to *Myriopteris*, we revert to the older epithet.

**7) ***Myriopteris clevelandii* (D. C. Eaton) Grusz & Windham, **comb. nov.** *Cheilanthes clevelandii* D. C. Eaton, Bull. Torrey Bot. Club 6: 33. 1875. Type: USA. California: Growing on a mountain about forty miles from San Diego at an elevation of about 2500 feet, Cleveland s.n. (holotype: YU; isotypes: GH, P, US). urn:lsid:ipni.org:names:77134846-1

**8) ***Myriopteris cooperae* (D. C. Eaton) Grusz & Windham, **comb. nov.** *Cheilanthes cooperae* D. C. Eaton, Bull. Torrey Bot. Club 6: 33. 1875. Type: USA. California: near Santa Barbara, Mrs. Ellwood Cooper (syntype: YU); Sierra Valley, Lemmon s.n. (syntype: YU). urn:lsid:ipni.org:names:77134847-1

**9) ***Myriopteris covillei* (Maxon) Á. Löve & D. Löve, Taxon 26: 325. 1977. *Cheilanthes covillei* Maxon, Proc. Biol. Soc. Wash. 31: 147. 1918. Type: USA. California: Surprise Canyon, Panamint Mountains, 13 April 1891, 1550 meters, Coville & Funston 593 (holotype: US). urn:lsid:ipni.org:names:77134848-1

**10) ***Myriopteris cucullans* (Fée) Grusz & Windham, **comb. nov.** *Cheilanthes cucullans* Fée, Mém. Fam. Foug. 7: 39, t. 25, f. 4. 1857. Type: Mexico, ad vallem Mexicanum, Schaffner 82 [holotype: RB; isotypes: K, US (fragment)]. urn:lsid:ipni.org:names:77134873-1

**11) ***Myriopteris fendleri* (Hook.) E. Fourn., Mex. Pl. 1: 125. 1872. *Cheilanthes fendleri* Hook., Sp. Fil. 2: 103, p. 107b. 1852. Type: USA. New Mexico, 1847, Fendler 1015 [holotype: K; isotypes: GH, MO, NY, US (fragment)].

**12) ***Myriopteris × fibrillosa*(Davenp.) Grusz & Windham, **comb. nov.** *Cheilanthes lanuginosa* var. *fibrillosa* Davenp., Bull. Torrey Bot. Club 12: 21. 1885. *Cheilanthes fibrillosa* (Davenp.) Davenp., Bull. Torrey Bot. Club 15: 225. 1888. Type: USA. California: San Jacinto Mountains, June 1882, Parish & Parish s.n. (holotype: GH). urn:lsid:ipni.org:names:77134880-1

**13) ***Myriopteris fimbriata* (A. R. Sm.) Grusz & Windham, **comb. nov.** *Cheilanthes microphylla* (Sw.) Sw. var. *fimbriata* A. R. Sm., Amer. Fern J. 70: 19, 21., f. 9–10. 1980. Type: Mexico. Chiapas: Munic. Frontera Comalapa, 6–8 km east of Frontera Comalapa, Breedlove 39018 (holotype: DS). urn:lsid:ipni.org:names:77134881-1

* Cheilanthes fimbriata* (A. R. Sm.) Mickel & Beitel, Mem. New York Bot. Gard. 46: 112. 1988. *hom. illeg., non Cheilanthes fimbriata* Vis., Fl. Dalmat. 1. 42 t. 1 f. 1. 1842.

**14) ***Myriopteris gracilis* Fée, Mém. Fam. Foug. 5: 150, t. 29, f. 6. 1852. *Cheilanthes gracilis* (Fée) Mett. ex Riehl, Abh. Senckenberg. Naturf. Ges. 80. 1859. *hom. illeg., non Cheilanthes gracilis* (Michx.) Kaulf., Enum. Filic. 209. 1824. Type: USA. Missouri: Jefferson County, Habitat ad rupes circa Hillsboro, Americâ septentr., Riehl 529 (isotypes: MO, US).

* Cheilanthes feei* T. Moore, Index Fil., 38. 1857.

* Myriopteris lanuginosa* J. Sm. Hist. Fil. 280. 1875. [*non M. lanuginosa* (Mart. & Gal.) E. Fourn. Mexic. Pl. 1: 125. 1872.]

 In *Cheilanthes*, this has been called *Cheilanthes feei* T. Moore because use of the oldest applicable epithet (based on *Myriopteris gracilis* Fée) was blocked by the earlier publication of *Cheilanthes gracilis* (Michx.) Kaulf. With the transfer of this species to *Myriopteris*, we revert to the original name published by Fée in 1852.

**15)*** Myriopteris gracillima* (D. C. Eaton) J. Sm., Hist. Fil. 280. 1875. *Cheilanthes gracillima* D. C. Eaton, Rep. U.S. Mex. Bound. Botany 2: 234. 1859. Type: USA. Oregon: Cascade Mountains, 7000 feet of altitude, latitude 44°, Bigelow s.n. (lectotype: YU).

**16) ***Myriopteris intertexta* (Maxon) Grusz & Windham, **comb. nov.** *Cheilanthes covillei* Maxon subsp. *intertexta* Maxon, Proc. Biol. Soc. Wash. 31: 149. 1918. *Cheilanthes intertexta* (Maxon) Maxon in Abrams, Ill. Fl. Pacific States 1: 28. 1923. Type: USA. California: Santa Clara County, Santa Cruz Mountains, collected at the top of Black Mountain, 6 July 1903, Dudley s.n. (holotype: DS). urn:lsid:ipni.org:names:77134849-1

**17)*** Myriopteris jamaicensis* (Maxon) Grusz & Windham, **comb. nov.** *Cheilanthes jamaicensis* Maxon, Contr. U.S. Natl. Herb. 24: 51. 1922. Type: Jamaica. Below Cinchona, 28 February 1919, Harris 12905 (holotype: US; isotypes: GH, MO, NY). urn:lsid:ipni.org:names:77134850-1

**18) ***Myriopteris lanosa* (Michx.) Grusz & Windham, **comb. nov.** *Nephrodium lanosum* Michx. Fl. Bor.-Amer. 2: 270. 1803. *Cheilanthes lanosa* (Michx.) D. C. Eaton, Rep. U.S. Mex. Bound., Botany 2: 234. 1859. Type: USA. Tennassee (sic) et Carolinae septentrionalis (non designatus). urn:lsid:ipni.org:names:77134851-1

* Myriopteris vestita* (Sw.) J. Sm., Cul. Ferns 29. 1857. (fide C. Chr. 1906.) *Adiantum vestitum* Spreng., Anleit. Kenntn. Gew. 3: 122. 1804.

**19) ***Myriopteris lendigera* (Cav.) Fée, Mém. Fam. Foug. 5: 149. 1852 (as *Myriopteris lentigera*). *Pteris lendigera* Cav., Descr. Pl. 268. 1801. *Cheilanthes lendigera* (Cav.) Sw., Syn. Fil. 128, 328. 1806. Type: Mexico. Hidalgo: Ixmiquilpan en la Nueva España, Nee s.n. [syntype: MA, US (fragment)]; Ecuador. Bolivar: junto á Guaranda en el Reyno de Quito, Nee s.n. (syntype: MA).

* Cheilanthes minor* Mart. & Gal. Mém. Act. Brux. 75, pl. 21, f. 1. 1842. *Myriopteris minor* (Mart. & Gal.) Fée, Mém. Fam. Foug. 5: 150. 1852.

* Cheilanthes lanuginosa* Mart. & Gal. Mém. Act. Brux. 75, pl. 20, f. 2. 1842. *Myriopteris lanuginosa* (Mart. & Gal.) E. Fourn. Mex. Pl. 1: 125. 1872.

* Myriopteris villosa* Fée, Mém. Fam. Foug. 5: 149. t. 28, f. 1. 1852.

* Cheilanthes frigida* Linden ex T. Moore, Gard. Chr. 772. 1857. *Myriopteris frigida* (Linden ex T. Moore) J. Sm. Cat. Cult. Ferns 28. 1857.

* Myriopteris lendigera* (Cav.) J. Sm., Cat. Cult. Ferns 28. 1857. *hom. illeg*.

* Pomataphytum pocillatum* M. E. Jones, Contributions to Western Botany 16: 12. 1930.

**20) ***Myriopteris lindheimeri* (Hook.) J. Sm., Bot. Voy. Herald. 340. 1856. *Cheilanthes lindheimeri* Hook., Sp. Fil. 2: 101, t. 107a. 1852. Type: USA. Western Texas, 1847, Lindheimer 744 [lectotype: K; isolectotypes: GH, P (2 sheets), SD, US, YU].

**21) ***Myriopteris longipila* (Baker) Grusz & Windham, **comb. nov.** *Cheilanthes longipila* Baker, Ann. Bot. (Oxford) 5: 211. 1891. Type: Mexico. San Luis Potosí, 22°N Lat., 6000–8000 ft., Parry & Palmer 989 [holotype: K; isotype: US (fragment)]. urn:lsid:ipni.org:names:77134852-1

**22)*** Myriopteris longipila* subsp. *brevipila* (Mickel) Grusz & Windham, **comb. nov.** *Cheilanthes longipila* var. *brevipila* Mickel, Mem. New York Bot. Gard. 88: 198–199, f. 84N–Q, 87J–M. 2004. Type: Mexico. Guerrero: 2 km al SE de Amatitlán, 1600 m, 13 August 1994, Soto 1052 (holotype: NY; isotype: FCME). urn:lsid:ipni.org:names:77134882-1

**23)*** Myriopteris marsupianthes* Fée, Mém. Fam. Foug. 5: 149, t. 12A, f. 1. 1852. *Cheilanthes marsupianthes* (Fée) T. Reeves *ex* Mickel & A. R. Sm. Mem. New York Bot. Gard. 88: 201, f. 83M–P. 2004.Type: Mexico. Veracruz: Pic d’Orizaba, Martens & Galeotti 6256 (holotype: P; isotype: BR).

**24) ***Myriopteris maxoniana* (Mickel) Grusz & Windham, **comb. nov.** *Cheilanthes maxoniana* Mickel, Mem. New York Bot. Gard. 88: 201, f. 87A–D. 2004. Type: Mexico. Tamaulipas: San Lucas, Viereck 76 (holotype: US). urn:lsid:ipni.org:names:77134853-1

**25) ***Myriopteris mexicana* (Davenp.) Grusz & Windham, **comb. nov.** *Cheilanthes mexicana* Davenp., Bull. Torrey Bot. Club 15: 227. 1888. Type: Mexico. Chihuahua: on the verge of a high cliff near the summit of Potrero Peak (Santa Eulalia Mts.), October 1886, 7300 ft., Pringle 827 (holotype: GH; isotypes: MO, BR, DS, NY, P, UC, US, YU). urn:lsid:ipni.org:names:77134854-1

**26) ***Myriopteris mickelii* (T. Reeves) Grusz & Windham, **comb. nov.** *Cheilanthes mickelii* T. Reeves, Brittonia 32: 502, f. 1–5. 1980. Type: Mexico. Oaxaca: Distr. Yautepec, Mickel 4210 (holotype: NY; isotypes: MO, UC). urn:lsid:ipni.org:names:77134855-1

**27) ***Myriopteris microphylla* (Sw.) Grusz & Windham, **comb. nov.** *Adiantum microphyllum* Sw., Prodr. 135. 1788. *Cheilanthes microphylla* (Sw.) Sw., Syn. Fil. 127. 1806. Type: Jamaica, Swartz s.n. (holotype: S). urn:lsid:ipni.org:names:77134856-1

**28) ***Myriopteris moritziana* (Kunze) Grusz & Windham, **comb. nov.** *Cheilanthes moritziana* Kunze, Linnaea 23: 307. 1850. Type: Venezuela. Caracas: La Guayra, Moritz 263 (lectotype: B; isolectotype: GH). urn:lsid:ipni.org:names:77134857-1

**29) ***Myriopteris myriophylla* (Desv.) J. Sm., Bot. Voy. Herald, 340. 1856. *Cheilanthes myriophylla* Desv., Ges. Naturf. Freunde Berlin Mag. Neuesten Entdeck. Gesammten Naturk. 5: 328. 1811. Type: South America. Anon. s.n. (holotype: P).

* Cheilanthes elegans* Desv. Ges. Naturf. Freunde Berlin Mag. 5: 328. 1811. *Myriopteris elegans* (Desv.) J. Sm., Cat. Cult. Ferns 29. 1857.

* Cheilanthes paleacea* Mart. & Gal., Mém. Foug. Mexique 76, pl. 21, f. 2. 1842. *Myriopteris paleacea* (Mart. & Gal.) Fée, Mém. Fam. Foug. 5: 149, t. 29, f. 6. 1852.

* Myriopteris intermedia* E. Fourn., Bull. Soc. Bot. Fr. 27: 328. 1880. *hom. illeg., non* Fée, Mém. Fam. Foug. 5: 149. 1852.

**30) ***Myriopteris newberryi*(D. C. Eaton) Grusz & Windham, **comb. nov.** *Notholaena newberryi* D. C. Eaton, Bull. Torrey Bot. Club 4: 12. 1873. *Cheilanthes newberryi* (D. C. Eaton) Domin, Biblioth. Bot. 20: 133. 1913. Types: USA. California: San Diego, 9 November 1857, Newberry 1352 (syntype: MO, YU); San Diego, 1866, Wood s.n. (syntype: YU); Southern California: S. W. corner of San Bernardino County, rocks in the Temescal range, 22 January 1861, W. H. Brewer s.n. (syntype: YU). urn:lsid:ipni.org:names:77134858-1

**31) ***Myriopteris notholaenoides* (Desv.) Grusz & Windham, **comb. nov.** *Pteris notholaenoides* Desv., Mém. Soc. Linn. Paris 6: 299. 1827. *Cheilanthes notholaenoides* (Desv.) Maxon *ex* Weath., Contr. Gray Herb. 114: 34. 1936. Type: Hispaniola, Anon. s.n. (holotype: P). urn:lsid:ipni.org:names:77134859-1

**32) ***Myriopteris × parishii* (Davenp.) Grusz & Windham, **comb. nov.** *Cheilanthes parishii* Davenp., Bull. Torrey Bot. Club 8: 59. 1881. Type: USA. California: San Diego County, W. J. Parish s.n. (holotype: GH; isotypes: GH, YU). urn:lsid:ipni.org:names:77134860-1

**33) ***Myriopteris parryi* (D. C. Eaton) Grusz & Windham, **comb. nov.** *Notholaena parryi* D. C. Eaton, Amer. Naturalist 9: 351. 1875. *Cheilanthes parryi* (D. C. Eaton) Domin, Biblioth. 85: 133. 1913. Type: USA. Utah: C. C. Parry 263 (holotype: YU; isotypes: GH, US, YU). urn:lsid:ipni.org:names:77134861-1

**34) ***Myriopteris peninsularis* (Maxon) Grusz & Windham, **comb. nov.** *Cheilanthes peninsularis* Maxon, Contr. U.S. Natl. Herb. 10: 496. 1908. Type: Mexico. Baja California, T. S. Brandegee s.n. (holotype: US). urn:lsid:ipni.org:names:77134862-1

**35) ***Myriopteris peninsularis*subsp. *insularis* (Weath.) Grusz & Windham, **comb. nov.** *Cheilanthes peninsularis* (Maxon) var. *insularis* Weath., Amer. Fern J.21: 25. 1931. Type: Mexico. Socorro Island, Mason 1616 (holotype: CAS). urn:lsid:ipni.org:names:77134884-1

**36) ***Myriopteris pringlei* (Davenp.) Grusz & Windham, **comb. nov.** *Cheilanthes pringlei* Davenp., Bull. Torrey Bot. Club 10: 61, t. 34. 1883. Type: USA. Arizona: C. G. Pringle s.n. (holotype: GH; isotypes: DS, MO, NY, US, YU). urn:lsid:ipni.org:names:77134863-1

**37)*** Myriopteris pringlei* subsp. *moncloviensis* (Baker) Grusz & Windham, **comb. nov.** *Cheilanthes moncloviensis* Baker, Ann. Bot. (Oxford) 5: 210. 1891. *Cheilanthes pringlei* var. *moncloviensis* (Baker) Mickel, Mem. New York Bot. Gard. 88: 207–208, f. 79J–M. 2004. Type: Mexico. Coahuila: Soledad, E. Palmer 1378 (holotype: K; isotypes: MO, NY, US). urn:lsid:ipni.org:names:77134864-1

**38) ***Myriopteris rawsonii*(Mett. ex. Kuhn) Grusz & Windham, **comb. nov.** *Cheilanthes rawsonii* Mett. ex. Kuhn, Filices Africanae 75. 1868. Type: Africa. Cape Province: Namaqualand, between Specktakel and Komaggas, Whitehead s.n. (holotype: BM; isotype: K). urn:lsid:ipni.org:names:77134878-1

**39) ***Myriopteris rufa*Fée, Mém. Fam. Foug. 8: 77. 1857. Type. Mexico. Veracruz: Volcan de Orizaba, Schaffner 83 (holotype: P?; isotype: RB?).

* Cheilanthes eatonii* Baker in Hook. & Baker, Syn. Fil. 140. 1867.

* Cheilanthes castanea* Maxon, Proc. Biol. Soc. Wash. 32: 111. 1919.

 In *Cheilanthes*, this has been called *Cheilanthes eatonii* Baker. Examination of putative type specimens of *Myriopteris rufa* housed at RB (digital image) and P indicates that the latter name very likely represents the same species as broadly defined by recent authors (e.g., Mickel and Smith 2004). Because *Myriopteris rufa* (published in 1857) has priority over *Cheilanthes eatonii* (1867), we take up Fée’s original name for this taxon in *Myriopteris*.

**40) ***Myriopteris scabra* (C. Chr.) Grusz & Windham, **comb. nov.** *Pellaea scabra* C. Chr., Index Filic. 483. 1906. Type: USA. Texas: crevices of rock on hills, Turkey Creek, 25 June 1849, Wright 824 (holotype: K; isotypes: GH, NY, US).

* Cheilanthes aspera* Hook., Sp. Fil. 2: 111, t. 108A. 1852. *hom. illeg*., *non Cheilanthes aspera* Kaulf., Linnaea 6(1): 186. 1831. urn:lsid:ipni.org:names:77134865-1

* Cheilanthes horridula* Maxon, Amer. Fern J. 8: 94. 1918.

 In *Cheilanthes*, this has been called *Cheilanthes horridula* Maxon because use of the oldest legitimate epithet (based on *Pellaea scabra* C. Chr.) was blocked by the earlier publication of *Cheilanthes scabra* H. Karst. (Maxon 1918). With the transfer of this species to *Myriopteris*, we revert to the older, exceedingly appropriate epithet.

**41) ***Myriopteris tomentosa* (Link) Fée, Mém. Fam. Foug. 5: 149. 1852. *Cheilanthes tomentosa* Link, Hort. Berol. 2: 42. 1833. Type: Mexico. Anon. s.n. [holotype: B; isotypes: PH, US (fragment)].

* Cheilanthes bradburii* Hook., Sp. Fil. 2: 97, t. 109b. 1852. *Myriopteris bradburii* (Hook.) J. Sm. Hist. Fil. 280. 1875.

**42) ***Myriopteris viscida*(Davenp.) Grusz & Windham, **comb. nov.** *Cheilanthes viscida* Davenp., Bull. Torrey Bot. Club 6: 191. 1877. Types: USA. California: Eastern slope of the Sierra Nevada near San Gogorio Pass, April 1876, Parry & Lemmon 427 (syntype: NY); California/Nevada: Downieville Buttes and bluffs of White Water River on the Colorado Desert, April–May, Lemmon s.n. (syntype: NY). urn:lsid:ipni.org:names:77134866-1

**43) ***Myriopteris windhamii* Grusz, Amer. Fern J. 103: 113. 2013. Type: USA. Arizona: Huachuca Mountains, Windham 4165 (holotype: DUKE; isotypes: ARIZ, ASC, ASU, GH, MO, NMC, NY, TEX/LL, UNM, US, UT).

* Cheilanthes villosa* Davenp. *ex* Maxon, Proc. Biol. Soc. Wash. 31: 142. 1918.

 In *Cheilanthes*, this has been called *Cheilanthes villosa* Davenp. *ex* Maxon. Because transfer of the epithet *villosa* to *Myriopteris* is blocked by the earlier publication of *Myriopteris villosa* Fée (= *Myriopteris lendigera* fide Reeves 1979), we use the replacement name for this distinctive taxon published by Grusz (2013).

**44)*** Myriopteris wootonii* (Maxon) Grusz & Windham, **comb. nov.** *Cheilanthes wootonii* Maxon, Proc. Biol. Soc. Wash. 3: 146. 1918. Type: USA. Arizona: Santa Rita Mountains, Wooton s.n. (holotype: US). urn:lsid:ipni.org:names:77134867-1

**45) ***Myriopteris wrightii* (Hook.) Grusz & Windham, **comb. nov.** *Cheilanthes wrightii* Hook., Sp. Fil. 2: 87, t. 110A. 1858. Type: USA. Texas–New Mexico: Wright 823 (holotype: K; isotypes: GH, NY, US). urn:lsid:ipni.org:names:77134868-1

**46)*** Myriopteris yatskievychiana* (Mickel) Grusz & Windham, **comb. nov.** *Cheilanthes yatskievychiana* Mickel, Mem. New York Bot. Gard. 88: 212–213, f. 74F–K. 2004. Type: Mexico. Sonora: Sierra del Aliso, A. Búrquez M. 96-302 (holotype: MO). urn:lsid:ipni.org:names:77134869-1

**47) ***Myriopteris yavapensis* (T. Reeves *ex* Windham) Grusz & Windham, **comb. nov.** *Cheilanthes yavapensis* T. Reeves *ex* Windham, Contr. Univ. Michigan Herb. 19: 32. 1993. Type: USA. Arizona: Yavapai County, Windham 202 (holotype: UT; isotypes: ASC, ASU, US). urn:lsid:ipni.org:names:77134879-1

**Name of uncertain application**

***Myriopteris cheiloglyphis***Fée, Mém. Fam. Foug. 8: 77. 1857.

**Excluded names**

***Myriopteris contracta***(Kunze) Fée, Mém. Fam. Foug. 5: 149. 1852. = *Cheilanthes contracta* (Kunze) Mett. ex Kuhn

***Myriopteris hirta***(Sw.) J. Sm., Ferns Brit. and For. 174. 1866. = *Cheilanthes hirta* Sw.

***Myriopteris induta*** (Kunze) Fée, Mém. Fam. Foug. 5: 149. 1852. = *Cheilanthes induta* Kunze

***Myriopteris intermedia***(Kunze) Fée, Mém. Fam. Foug. 5: 149. 1852. = *Cheilanthes hirta* Sw. fide Christensen (1906)

***Myriopteris macleanii***J. Sm., Hist. Fil. 280. 1875. = *Cheilanthes pilosa* Goldm. fide Christensen (1906)

***Myriopteris scariosa***(Sw.) Fée, Mém. Fam. Foug. 5: 149, t. 29, f. 6. 1852. = *Cheilanthes scariosa* Sw.

***Myriopteris szovitzii***(Fisch. & Meyer) J. Sm., Hist. Fil. 281. 1875. = *Cheilanthes persica* (Bory) Mett. ex Kuhn fide Christensen (1906)

## Supplementary Material

XML Treatment for Myriopteris
